# Addition of Nano CaF_2_@SiO_2_ and SiC Whiskers in Ceramic Tools for Wear Reduction and Improved Machinability

**DOI:** 10.3390/ma15155430

**Published:** 2022-08-07

**Authors:** Wenhao Zhang, Zhaoqiang Chen, Congfeng Tian, Jun Wu, Guangchun Xiao, Niansheng Guo, Mingdong Yi, Jingjie Zhang, Chonghai Xu

**Affiliations:** 1 School of Mechanical Engineering, Qilu University of Technology (Shandong Academy of Sciences), Jinan 250353, China; 2 Shandong Machinery Design and Research Institute, Qilu University of Technology (Shandong Academy of Sciences), Jinan 250031, China; 3 Key Laboratory of Advanced Manufacturing and Measurement and Control Technology for Light Industry in Universities of Shandong, Qilu University of Technology (Shandong Academy of Sciences), Jinan 250353, China; 4 Shantui Construction Machinery Co., Ltd., Jining 272035, China

**Keywords:** nanoparticles, whisker, ceramic, wear reduction, cutting performance

## Abstract

The addition of CaF_2_@SiO_2_ and SiC whiskers to ceramic tools can improve their flexural strength and fracture toughness, reduce surface damage, and improve their cutting performance. The cutting experiments showed that under the same cutting conditions, the surface roughness of the workpiece processed with the Al_2_O_3_/TiC/SiC/CaF_2_@SiO_2_ (ATSC10) tool was significantly lower than that of the workpiece processed with the Al_2_O_3_/TiC/ SiC (ATS) tool. Additionally, the main cutting force and cutting temperature when cutting with the ATSC10 tool were lower by 30 and 31.7%, respectively. These results were attributed to the precipitation of CaF_2_ from the nanocoated particles during cutting and the formation of a uniform and continuous lubricating film on the surface of the tool. The wear on the front surface of the ATS tool was mainly adhesive, and that on the back tool surface was mainly abrasive. For ATSC10, the main forms of wear on the tool front surface were adhesive and abrasive, whereas the main form of wear on the tool back surface was abrasive with slight adhesive wear. The addition of nano-coated particles and whiskers improved the mechanical properties of the cutting tool while maintaining good cutting performance.

## 1. Introduction

High-speed dry cutting is an important processing method that does not make use of cutting fluids [1]. Ceramic cutting tools are widely used because of their advantages of high-temperature resistance, good wear resistance, high hardness, good chemical stability, low affinity for the processed workpiece material, and considerable resistance to producing chip tumors [2,3,4]. However, low flexural strength [5] and low fracture toughness [6] in ceramic materials lead to failure, such as edge breakage and fracture [7], when ceramic tools are subjected to high temperatures and high impact stress [8] during high-speed dry cutting.

To meet the requirements of developing high-speed dry cutting, users tend to add solid lubricants to ceramic tools to reduce the negative impact of not making use of cutting fluids to realize the self-lubricating effect of props [9,10,11,12]. However, the addition of a solid lubricant affects the mechanical properties of the tool itself [13], so achieving a good balance between the self-lubricating function and mechanical properties is the goal that people have been seeking to meet.

Whisker toughening is currently an ideal toughening method for ceramic materials [14,15]. It has a good toughening effect through the toughening mechanisms of whisker pulling [16], crack deflection [17], and whisker bridging [18]. Lao et al. [19] strengthened glass ceramics by forming SiC whiskers in situ, which significantly reduced the thermal expansion coefficient of glass ceramics and improved their mechanical properties. The flexural strength of the glass ceramics reached a maximum value of 75.7 MPa at the maximum content of in situ SiC whiskers. Fang et al. [20] prepared a ZrO_2_ whisker-reinforced titanium carbonitride Ti(C, N)-based cermet tool material. The material with 7.5 wt.% ZrO_2_ whiskers had 32.5% higher fracture toughness and flexural strength compared with the ceramic tool material without ZrO_2_ whiskers; however, its relative density was lower.

Compared to the single toughening method, the compound toughening method has a higher toughening effect [21]. Li et al. [22] prepared a composite ceramic tool with calcium fluoride-coated particles and zirconia whiskers simultaneously, which exhibited a toughening superposition effect: the flexural strength and fracture toughness of the composite ceramic materials increased by 10 and 11%, respectively, compared with the zirconia whisker-toughened ceramic materials. Zhao et al. [23] studied the effect of the addition of nano TiC particles on SiC whisker-toughened Al_2_O_3_ ceramics. They found that at a nano TiC particle size of 40 nm and a content of 4 vol%, the comprehensive mechanical properties of the ceramic materials were the best. Chen et al. [24] found that the simultaneous addition of surface-modified calcium fluoride and zirconia whiskers to ceramic tools could improve their fracture toughness, realize the self-lubricating effect of props, and achieve a good balance between the self-lubricating function and mechanical properties.

Therefore, in this study, the effects of the addition of SiC whiskers and CaF_2_@SiO_2_ nano-coated particles to a ceramic tool material on the mechanical properties and microstructure were tested. Methods for reducing the friction coefficient and improving the wear resistance of cutting tools have been studied through friction and wear tests. The cutting parameters, cutting force, cutting temperature, and tool wear degree during cutting were studied experimentally. The wear reduction mechanism of nano-CaF_2_@SiO_2_ and SiC whiskers in ceramic tools was discussed and analyzed.

## 2. Experimental

### 2.1. Preparation of a Self-Lubricating Tool

In this study, the coated particles (CaF_2_@SiO_2_) with core–shell shape were obtained by coating the surface of calcium fluoride with a layer of silica. Al_2_O_3_/TiC/SiC (ATS) and Al_2_O_3_/TiCSiC//CaF_2_@SiO_2_ (ATSC10, where 10 represents the volume content of CaF_2_@SiO_2_) self-lubricating ceramic tool materials were prepared via vacuum hot-pressing sintering. The specific compositions of the ceramic materials are listed in Table 1. Table 2 lists the basic information on the raw materials.

The specific preparation process of ceramic cutting tool materials is as follows.

Each component material was weighed according to the component ratio listed in Table 1. An appropriate amount of absolute ethyl alcohol was added to the weighed Al_2_O_3_, TiC, and MgO powders. The component suspensions were individually ultrasonicated for 25 min, after which they were mixed and ultrasonicated for 25 min. The dispersed suspension was transferred into a ball mill and milled for 44 h at a grinding balls to ceramic materials ratio of 10:1. To prevent oxidation, milling was conducted under a nitrogen atmosphere. Absolute ethyl alcohol and polyethylene glycol were added to the weighed nano-coated particles and whiskers; then, the suspensions were ultrasonicated and added to a ball milling tank, and the mixed suspension was ball-milled for 4 h. The obtained suspension was dried in a vacuum oven at 110 °C for 24 h. The dried powder was screened using a 200-mesh sieve, cold-pressed in a graphite mold, sintered in a vacuum hot-pressing sintering furnace, and ball-milled for 4 h.

**Figure 1:**
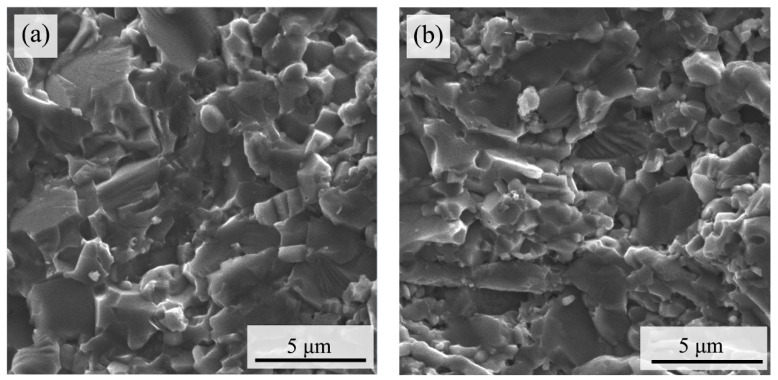

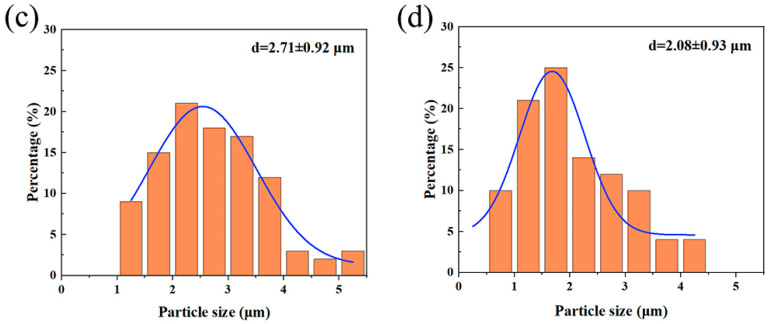
SEM images of the two ceramic cutting tools (**a**) ATS, (**b**) ATSC10; and particle size distribution (**c**) ATS and (**d**) ATSC10. The blue line is a Gaussian fitting curve, which reflects the general trend of particle size distribution.

Figure 1a,b shows the SEM images of the two ceramic cutting tools. Figure 1c,d shows the average particle sizes of the two ceramic cutting tools. ATS tool materials have an average grain size of 2.71 μm and ATSC10 has an average grain size of 2.08 μm. This indicates that simultaneously adding CaF_2_@SiO_2_ and SiC can greatly reduce the grain size of the ceramic cutting tools. The particle size of ATSC10 is significantly lower than that of ATS. Table 3 lists the mechanical properties of the ceramic materials. It is obvious that although the hardness of ATSC10 self-lubricating ceramic tools was lower than that of ATS ceramic tools, it was still high. At the same time, the ATSC10 self-lubricating ceramic tool had better flexural strength and fracture toughness.

### 2.2. Ceramic Tool Friction and Wear Test

The friction and wear performance of self-lubricating ceramic tools was experimentally studied using a UMT-2 friction and wear tester. The tests were performed under dry-sliding ball–surface reciprocating friction and wear. The experimental block was an Al_2_O_3_/TiC/SiC/CaF_2_@SiO_2_ self-lubricating ceramic tool with a cross-sectional area of 20 × 10 mm^2^. The friction ball was made of 45 steel with an inner diameter of 3 mm. The friction coefficient of Al_2_O_3_/TiC/SiC/CaF_2_@SiO_2_ was tested at a fixed friction velocity, load, and testing time of 100 mm/s, 20 N, and 30 min, respectively. Scanning electron microscopy (SEM) was utilized to observe the friction and wear morphologies of the self-lubricating ceramic tools to further elucidate their wear-reduction mechanisms.

### 2.3. Cutting Test of Ceramic Tool

ATS ceramic tools and ATSC10 self-lubricating ceramic tools were tested on a CDE6140A lathe, produced by Dalian Machine Tool Group Co. Ltd. (Dalian, China), and a Kenna GSSNR/L2525M12-MN7 tool shank. The tool flank wear *VB* was measured using a PXS-1020 tool microscope. When the *VB* reached 0.3 mm the tool was considered invalid, and the cutting test was stopped. The surface roughness of the machined workpiece was measured using a TR200 handheld roughness meter. The cutting temperature and cutting force were measured using an FLAR-A320 infrared thermal imager and a Kistler-9129A dynamometer. The workpiece was made of quenched and tempered 40Cr steel (38–44 HRC). Table 4 lists the geometric parameters of the cutting tool used in the cutting experiment.

## 3. Results and Discussion

### 3.1. Friction and Wear Properties of Self-Lubricating Tools

**Figure 2:**
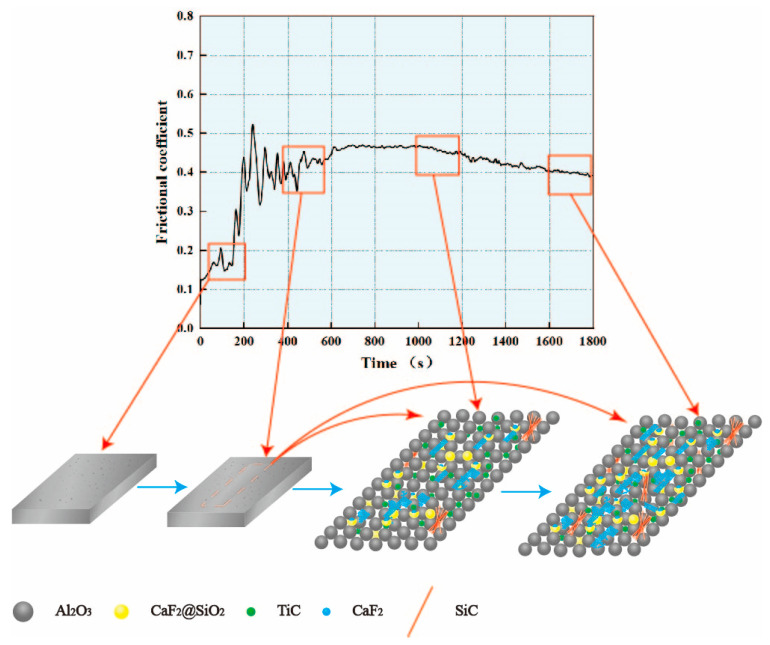
Friction coefficient over time.

Figure 2 shows a graph of the change in the friction coefficient with time. The friction coefficient is relatively small during the initial stage of the friction and wear experiments. This is because, although the self-lubricating ceramic tool was polished before the friction and wear experiment, its surface was not completely smooth and still contained many microscopic pits and protrusions. Therefore, at the initial stage of the experiment, only the raised plane on the tool surface came into contact with the experimental friction ball, leading to the actual contact surface being much smaller than the apparent contact area. The friction surface contact first occurred on a higher microconvex body. In the friction experiment, owing to stress concentration, the surface contact convex points gradually produced wear debris. This reduced the number of surface protrusions, smoothened the contact surface between the experimental friction ball and the self-lubricating ceramic tool, increased the friction area, and gradually changed the wear from point contact to small-surface contact. Therefore, the friction coefficient of the material fluctuated and increased during the initial wear stage. After approximately 600 s of friction and wear, the roughness of the material surface tended to stabilize. In addition, the wear of the material entered a relatively stable state, and both the friction factor curve and friction factor were relatively stable. After approximately 1000 s of friction and wear, the amount of wear on the material increased. At that time, because of the increase in wear time, the SiO_2_ coating layer of CaF_2_@SiO_2_ nano-coated particles broke because of wear, which caused the CaF_2_ nanoparticles to flow out and lubricate the tool. Moreover, the ATSC10 self-lubricating ceramic tool had excellent Vickers hardness flexural strength and fracture toughness, showing that the ATSC10 ceramic tool would not easily crack from surface damage and reduced the generation of wear debris; the surface was easier to keep smooth. The friction coefficient between the experimental friction ball and the tool surface was reduced by the effect of these two aspects until it stabilized at a smaller value.

**Figure 3:**
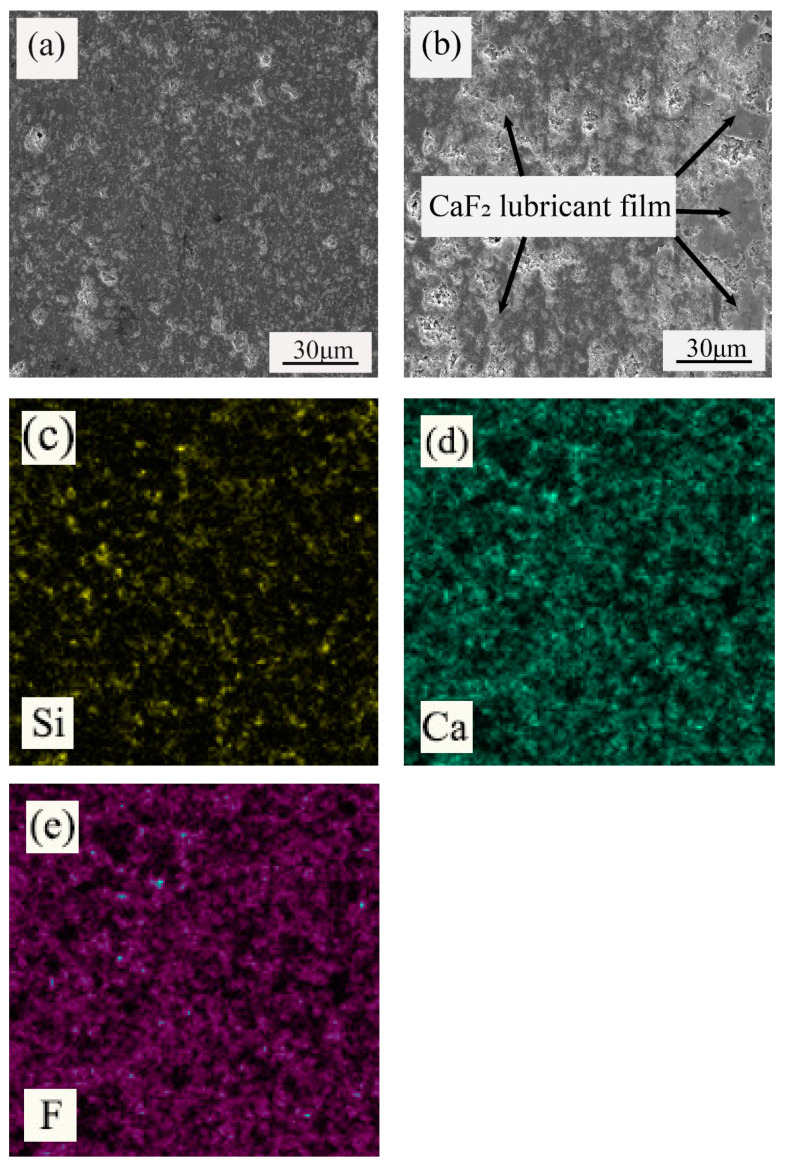
The wear morphology of the ATSC10 ceramic tools and elemental mapping: (**a**) the tool material before the friction and wear experiment, (**b**) the morphology of the tool material after the friction and wear experiment, (**c**) distribution of Si element, (**d**) distribution of Ca element, (**e**) distribution of F element.

The cross-sectional morphologies of the test blocks (which were ultrasonically cleaned and cut) were observed using SEM before and after the friction and wear experiments. Figure 3a shows the morphology of the tool material before the friction and wear experiment, whereas Figure 3b shows the morphology of the tool material after the friction and wear experiment. In Figure 3b, a continuous and uniform lubricating film can be observed on the tool surface, and an elemental analysis was performed. Figure 3c shows the distribution of the Si element, Figure 3d shows the distribution of the Ca element, and Figure 3e shows the distribution of the F element. It can be seen that the elements of Si, Ca, and F are evenly distributed in the tool material. Considering that Ca element and F element are from CaF_2_@SiO_2_, but Si is from CaF_2_@SiO_2_ and SiC, we found that the distribution of Ca and F is roughly the same, and the distribution of Ca and F elements is denser and more extensive than that of Si elements. Therefore, we can say that during the process of wear, CaF_2_ was separated, forming a uniform and continuous lubricating film on the surface of the tool, resulting in denser and more extensive distribution of Ca and F elements than Si elements, which proves the existence of the CaF_2_ lubrication film.

The above experimental studies show that Nano-CaF_2_@SiO_2_ and SiC whiskers can effectively reduce the friction coefficient of ceramic tool materials and achieve good wear-reduction performance.

### 3.2. Cutting Performance of Self-Lubricating Tools

#### 3.2.1. Effects of Cutting Parameters

Choosing proper cutting parameters can ensure good cutting performance of the ceramic tool during cutting.

**Figure 4:**
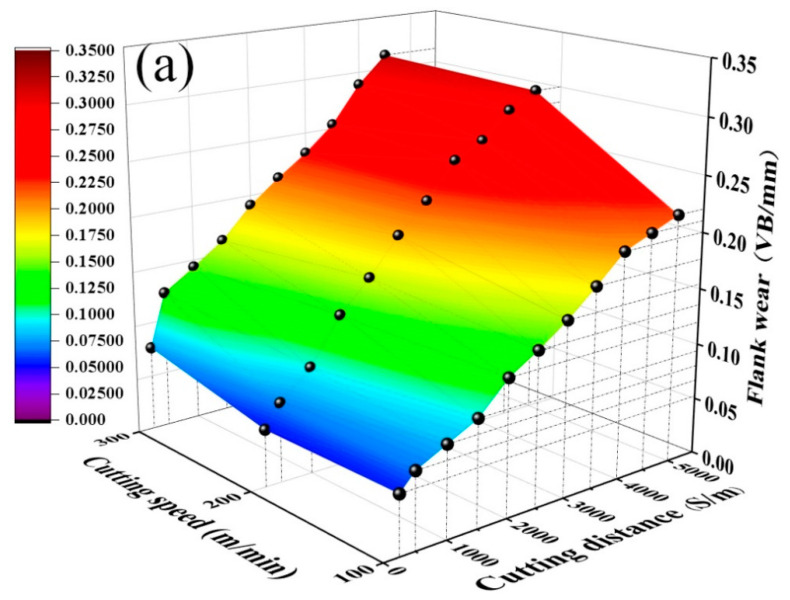

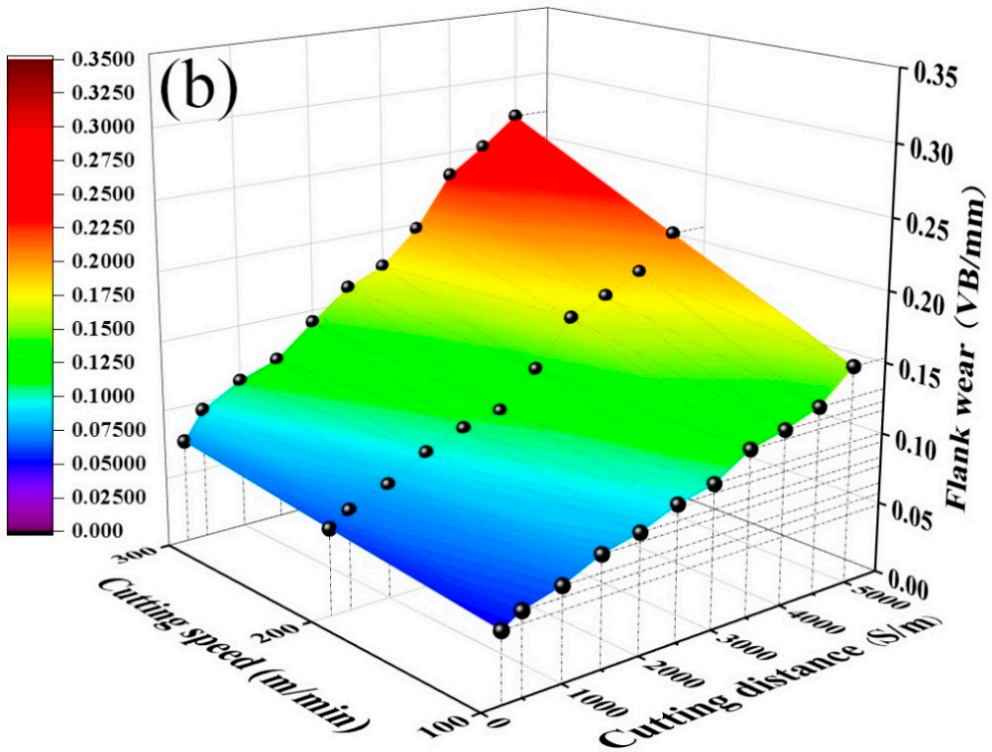
Curves of flank wear *VB* against cutting distance *S* under different cutting speeds: (**a**) ATS, (**b**) ATSC10.

Figure 4 shows that the flank wear of the ATS ceramic tool and ATSC10 self-lubricating ceramic tool follow approximately the same trend at different cutting speeds (cutting feed *f* = 0.102 mm/r, cutting depth *ap* = 0.2 mm). As shown in Figure 4a, at the cutting speed of 200 m/min, the ATS ceramic tool failed at 5000 m. At the cutting speed of 300 m/min, the ATS ceramic tool failed at 4500 m. As shown in Figure 4b, at the cutting distance of *S* = 5000 m and under different cutting speeds, the *VB* of the ATSC10 self-lubricating ceramic tools reached neither 0.3 mm nor the maximum cutting distance of the ceramic tools. The comparison of the two ceramic tools showed that the flank wears of the two types of tools followed approximately the same trend at different cutting speeds, but the increase in wear of the ATSC10 self-lubricating ceramic tool was small, and the effective cutting distance at different cutting speeds was larger than that of the ATS ceramic tool. This is because, although the hardness of ATSC10 self-lubricating ceramic tools was lower than that of ATS ceramic tools, it was still high. At the same time, the ATSC10 self-lubricating ceramic tool had better flexural strength, fracture toughness, and impact resistance, indicating that the ATSC10 ceramic tool would not easily crack during processing or from surface damage. When the cutting force was applied to the ATSC10 ceramic tool, the nano-coated particles were damaged, and CaF_2_, which has a low shear strength was separated, forming a uniform and continuous lubricating film on the surface of the tool. Consequently, the ATSC10 ceramic cutting tools exhibited less wear.

**Figure 5:**
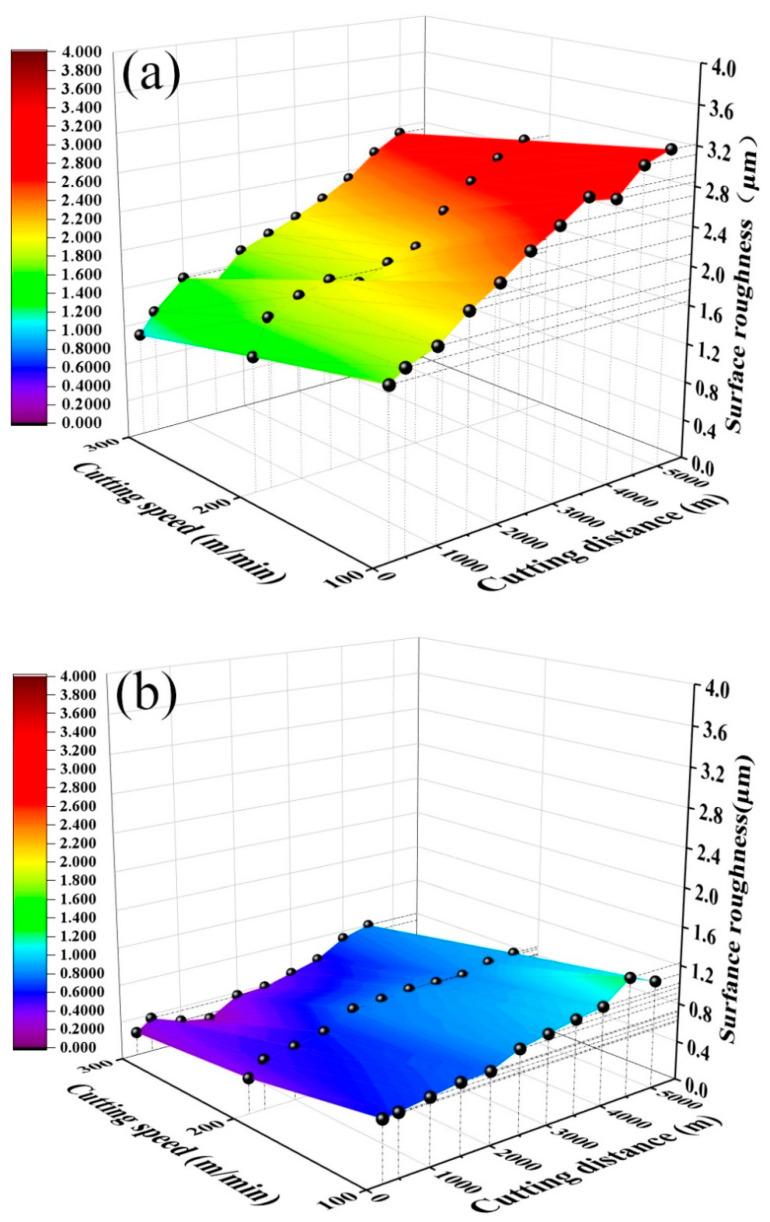
Workpiece surface roughness *R_a_* against cutting distance *S* at different cutting speeds: (**a**) ATS, (**b**) ATSC10.

Figure 5 shows the curves of surface roughness *R_a_* of the quenched and tempered 40Cr steel cut with the ATS ceramic tool and ATSC10 self-lubricating ceramic tool with a cutting distance *S* under different cutting speeds (cutting feed *f* = 0.102 mm/r, cutting depth *ap* = 0.2 mm). As shown in Figure 5, the two tools exhibit the same trend in the effect of cutting speed on surface roughness. However, the maximum surface roughness *R_a_* of the workpiece machined with the ATSC10 self-lubricating ceramic tool was 1.23 μm, which is much lower than that 3.21 μm of the workpiece machined with the ATS ceramic tool. During cutting with the prepared self-lubricating tool, the ATSC10 ceramic tool should not easily crack or from surface damage and reduced the generation of wear debris; thus, the surface is easier to keep smooth. At the same time, CaF_2_ in the nano-coated particles precipitated to form a solid lubricating film. This reduced the surface roughness of the processed workpiece, and resulted in good surface processing quality.

**Figure 6:**
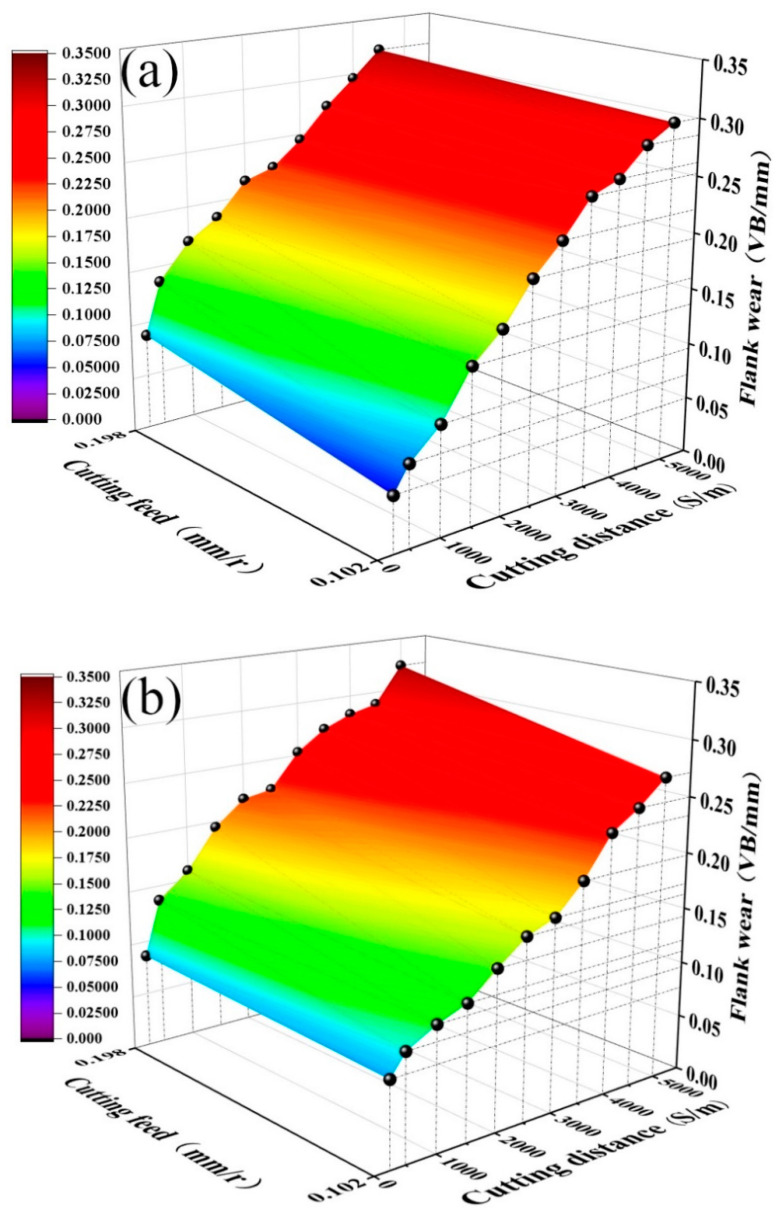
Curves of flank wear *VB* against cutting distance *S* under different cutting feed rates: (**a**) ATS, (**b**) ATSC10.

Figure 6 shows the curves of flank wear *VB* with cutting distance *S* under different cutting feed rates (cutting speed *v* = 300 m/min, cutting depth *ap* = 0.2 mm) when cutting quenched and tempered 40Cr steel with the ATS ceramic tool and ATSC10 self-lubricating ceramic tool. As shown in Figure 6, the flank wear of the ATS ceramic tool and the ATSC10 self-lubricating ceramic tool at *f* = 0.102 mm/r is smaller than at *f* = 0.198 mm/r. At *f* = 0.102 mm/r, the effective cutting distance of the ATSC10 self-lubricating ceramic tool exceeds 5000 m, while the effective cutting distance of the ATS ceramic tool is 5000 m. At *f* = 0.198 mm/r, the effective cutting distance of the ATSC10 self-lubricating ceramic tool is between 4500 and 5000 m, and the effective cutting distance of the ATS ceramic tool is less than 4500 m. With an increase in the cutting feed, the effective cutting distance of the two types of tools and their service life decreased. Additionally, with increasing cutting feed, the wear rate of the flank of the ATS ceramic tool increased more rapidly than that of the ATSC10 self-lubricating ceramic tool. Under different cutting feed rates, the effective cutting distances of the two types of tools were roughly the same. The addition of nano-coated particles and whiskers cause the ATSC10 ceramic cutting tools to exhibit less wear and improved the service life of ceramic cutting tools under different cutting feed rates.

**Figure 7:**
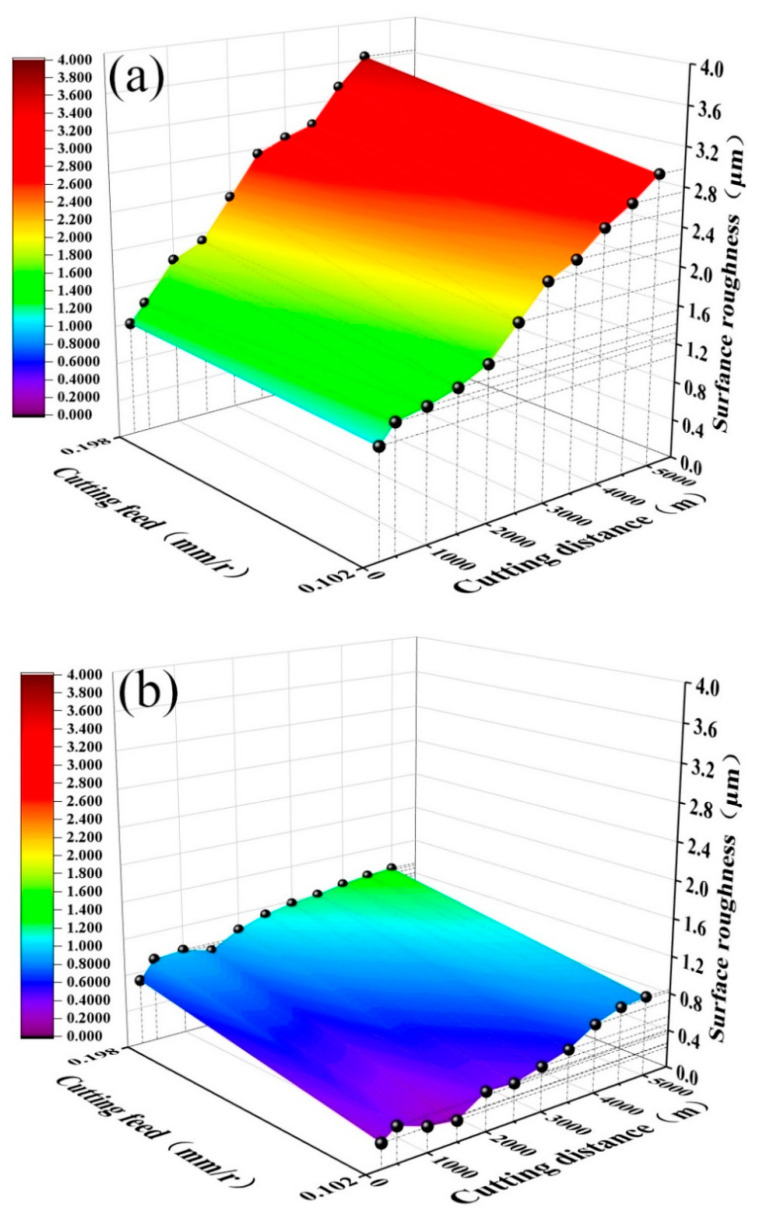
Workpiece surface roughness *R_a_* against cutting distance *S* under different cutting feed rates: (**a**) ATS, (**b**) ATSC10.

Figure 7 shows the curves of surface roughness *R_a_* of the quenched and tempered 40Cr steel cut with the ATS ceramic tool and the ATSC10 self-lubricating ceramic tool against cutting distance *S* under different cutting feed rates (cutting speed *v* = 300 m/min, cutting depth *ap* = 0.2 mm). With an increase in the cutting feed rate, the surface roughness *R_a_* of both workpieces increases with the feed rate; however, the surface roughness of the workpiece machined using the ATS ceramic tool is obviously larger than that of the workpiece machined with the ATSC10 self-lubricating ceramic tool. Based on the results, the feed rate of 0.102 mm/r may be considered optimal because, at this value, the surface roughness of the machined workpiece is low, and the effective cutting distance is large. The addition of nano-coated particles and whiskers cause the ATSC10 ceramic cutting tools to exhibit less wear, and improved the service life of ceramic cutting tools, reduced the surface roughness of the workpiece processed by the tool, and resulted in good surface processing quality under different cutting feed rates.

**Figure 8:**
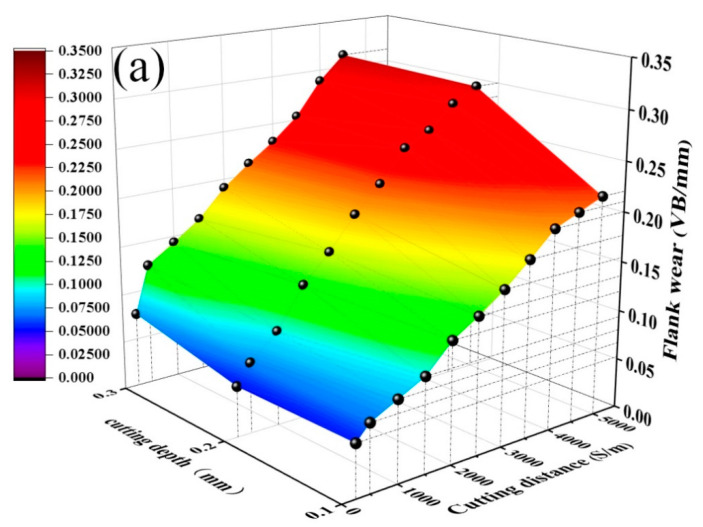

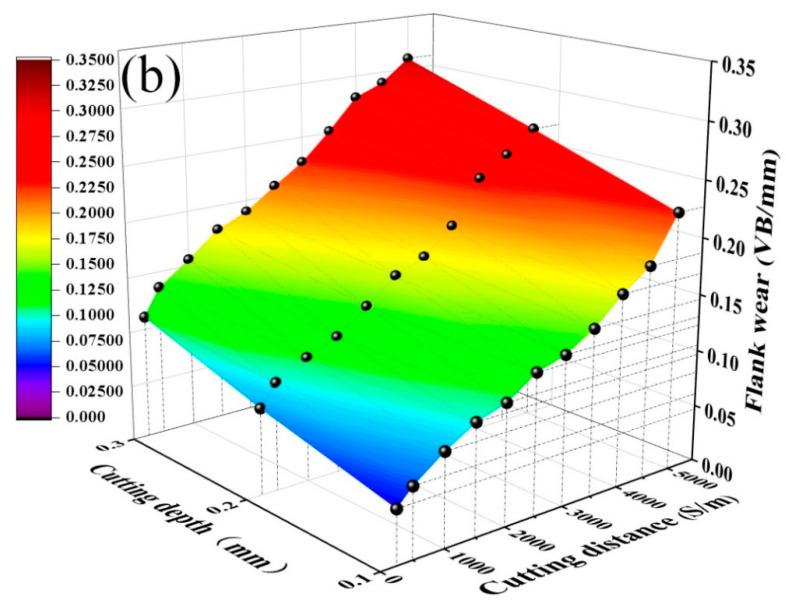
Curves of flank wear *VB* against cutting distance *S* at different cutting depths: (**a**) ATS, (**b**) ATSC10.

Figure 8 shows the curves of the flank wear *VB* of the quenched and tempered 40Cr steel cut with the ATS ceramic tool and the ATSC10 self-lubricating ceramic tool as a function of cutting distance *S* under different cutting depths (cutting speeds *v* = 300 m/min, cutting feed *f* = 0.102 mm/r). As shown in Figure 8a, the effective cutting distance of the ATS ceramic tool decreases with an increase in the cutting depth. At *ap* = 0.1 mm, the effective cutting distance of the ATS ceramic tool exceeds 5000 m. At *ap* = 0.2 mm, the effective cutting distance of the ATS ceramic tool is 5000 m. At *ap* = 0.3 mm and *S* = 4500 m, *VB* = 0.32 mm, and the effective cutting distance of the tool is less than 4500 m. As shown in Figure 8b, at *ap* = 0.1 and 0.2 mm, the effective cutting distance of the ATSC10 self-lubricating ceramic tool exceeds 5000 m. At *ap* = 0.3 mm and *S* = 5000 m, *VB* reaches 0.31 mm, and the effective cutting distance of the tool is between 4500 and 5000 m. The addition of nano-coated particles and whiskers cause the ATSC10 ceramic cutting tools to exhibit less wear and definitely improved the service life of ceramic cutting tools under different cutting depths.

**Figure 9:**
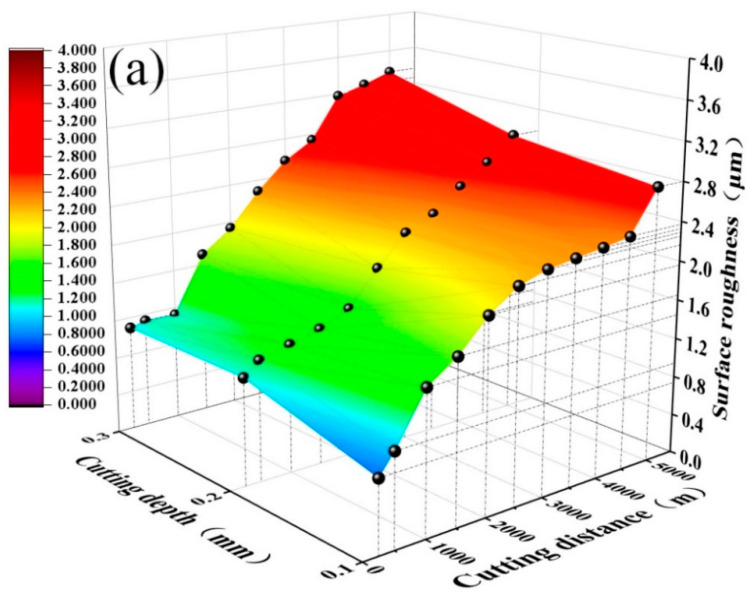

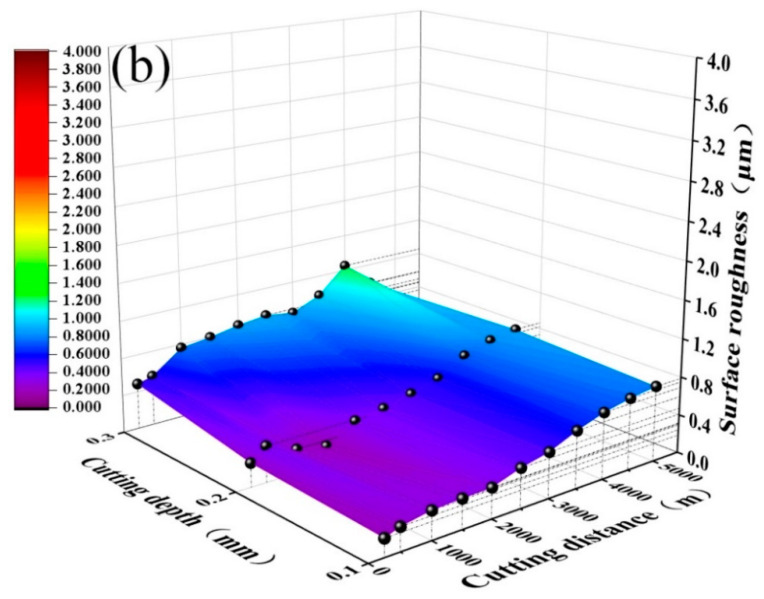
Workpiece surface roughness *R_a_* against cutting distance *S* at different cutting depths: (**a**) ATS, (**b**) ATSC10.

As shown in Figure 9, *R_a_* increases with cutting depth and distance for both ceramic tools. However, workpiece surface roughness for the ATS ceramic tool is obviously higher than that for the ATSC10 self-lubricating ceramic tool. The addition of nano-coated particles and whiskers caused the ATSC10 ceramic cutting tools to exhibit less wear, improved the service life of ceramic cutting tools, reduced the surface roughness of the workpiece processed by the tool, and resulted in good surface processing quality under different cutting depths. Based on the results, the back-cutting rate of 0.2 mm, at which the surface roughness of the machined workpiece is low, the effective cutting distance is large and the high cutting efficiency may be considered optimal.

In summary, the flank wear of ATS and self-lubricating ATSC10 ceramic tools increased with cutting speed, back-cutting rate, and feed rate. The surface roughness increased with the back-cutting rate and the feed rate and decreased with an increase in the cutting speed. The following optimal cutting parameters were determined: cutting speed of 300 m/min, feed rate of 0.102 mm/r, and back-cutting rate of 0.2 mm, at which time the surface roughness of the machined workpiece was low and the effective cutting distance was large and cutting was more efficient. Under different cutting parameters, the addition of nano-coated particles and whiskers make the service life of ATSC10 self-lubricating ceramic tools longer than that of ATS ceramic tools and, at the same time, reduced the surface roughness of the processed workpiece, and resulted in good surface processing quality.

#### 3.2.2. Effect of Nano-Coated Particles and Whiskers on Cutting Performance

##### Effect on Cutting Force

**Figure 10:**
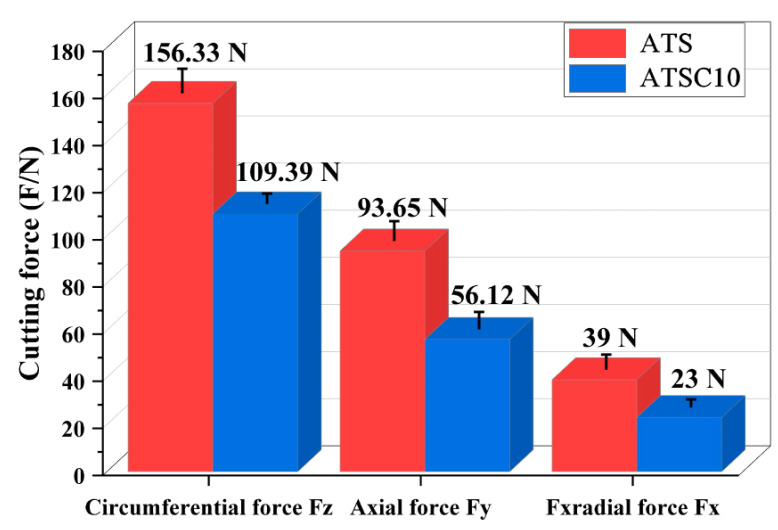
Comparison of cutting forces of the two types of tools.

Figure 10 shows a comparative diagram of the cutting forces of the ATS and ATSC10 ceramic tools under the same cutting parameters. The main cutting force *F_z_* for the ATSC10 ceramic tool is 30% lower than that for the ATS ceramic tool; the cutting depth resistance *F_x_* and feed force *F_y_* were also higher for ATS ceramic tools. This indicates that the simultaneous addition of CaF_2_@SiO_2_ and SiC whiskers significantly reduced the cutting force under the same cutting parameters, which resulted in better surface machining quality when using the ATSC10 ceramic tool.

##### Effect on Cutting Temperature

**Figure 11:**
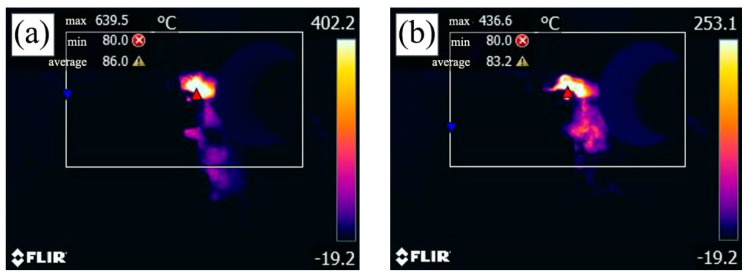
Cutting temperature diagram for the two cutting tools: (**a**) ATS, (**b**) ATSC10.

Figure 11 shows a comparison of the cutting temperatures of the ATS and ATSC10 ceramic tools at a cutting speed of 300 m/min after reaching a stable cutting stage. The other cutting parameters are as follows: cutting depth *ap* = 0.2 mm and feed rate *f* = 0.102 mm/r. As shown in Figure 11, the cutting temperature of the ATS ceramic tool under this test condition is 639.5 °C, and that of the ATSC10 ceramic tool is 436.6 °C. Thus, the cutting temperature of the ATSC10 ceramic tool is 31.7% lower. Thus, the addition of nano-coated particles effectively reduced the cutting temperature, which corresponds to the trend observed in the cutting force. CaF_2_ precipitated from the nano-coated particles to form a solid lubricating film, resulting in the ATSC10 ceramic tool having a lower cutting force and temperature during cutting than the ATS ceramic tool.

### 3.3. Mechanism Analysis

**Figure 12:**
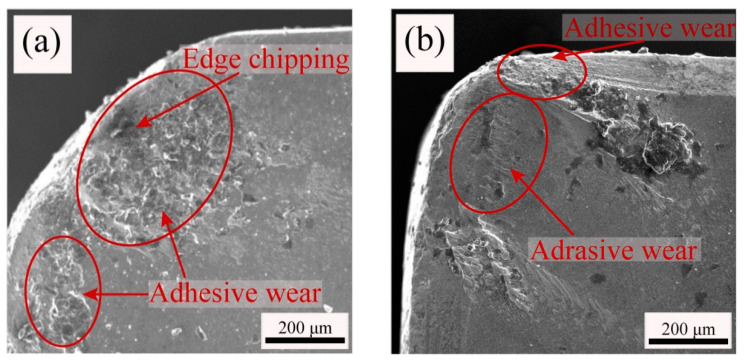

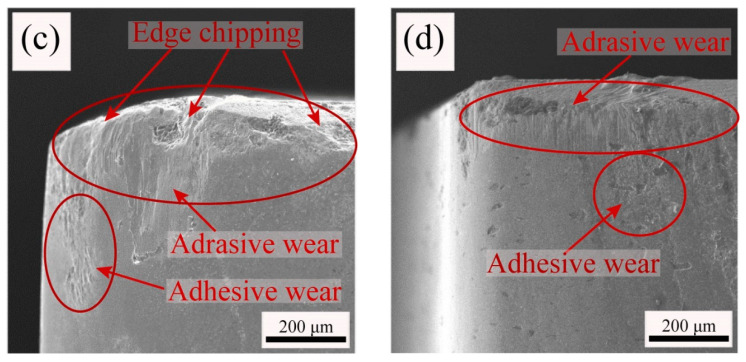
The wear morphology of the ATS and ATSC10 ceramic tools: (**a**) front surfaces of the ATS, (**b**) front surfaces of the ATSC10, (**c**) back face of the ATS, (**d**) back face of the ATSC10.

Figure 12a,b shows the wear morphologies of the front surfaces of the ATS and ATSC10 ceramic tools. Evidently, the bond wear of the ATS ceramic tool (Figure 12a) is significant and the front face of the ATS ceramic tool has a clearly chipped edge, whereas that of the ATSC10 ceramic tool (Figure 12b) is relatively light. At the same time, the ATSC10 ceramic tool front surface exhibits slight abrasive wear. 

This is because, during cutting, the ATS ceramic cutting tool generated a greater friction force between the cutter and workpiece, leading to gelling of the surfaces of the ceramic cutting tool and workpiece, resulting in the flow of the cutting tool material on the surface of the chip. But for ATSC10, although the hardness of ATSC10 self-lubricating ceramic tools was lower than that of ATS ceramic tools, it was still high. At the same time, the ATSC10 self-lubricating ceramic tool showed better flexural strength, fracture toughness, and impact resistance, indicating that the ATSC10 ceramic tool should not easily crack during processing or from surface damage. When the cutting force was applied to the ATSC10 ceramic tool, the nano-coated particles were damaged, and CaF_2_, which has a low shear strength was separated, forming a uniform and continuous lubricating film on the surface of the tool. Hence, the wear on the face of the ATS ceramic tools is mainly adhesive, the wear on the face of the ATSC10 ceramic tools is mainly slightly adhesive and abrasive.

Figure 12c,d show the back-tool surface wear morphologies of the ATS and ATSC10 ceramic tools. In Figure 12c, the back face of the ATS ceramic tool has a clearly chipped edge, and the edge of the back face is not smooth. The main reason for this is that when the ceramic cutting tool operates at a higher cutting speed, the cutting force is larger, thus causing damage to the back tool surface. Simultaneously, furrows caused by abrasive wear can be clearly observed on the wear surface. This is because, during cutting, a large cutting force between the tool and workpiece and high cutting temperature result in hard particles damaging the tool surface. Simultaneously, bond wear can be observed in the wear morphology of the rear tool. In Figure 12d, the ATSC10 ceramic tool wear mainly consists of abrasive and bond wear; during cutting, a small cutting force between the tool and workpiece and low cutting temperature reduced the hard particles damaging the tool surface.

**Figure 13:**
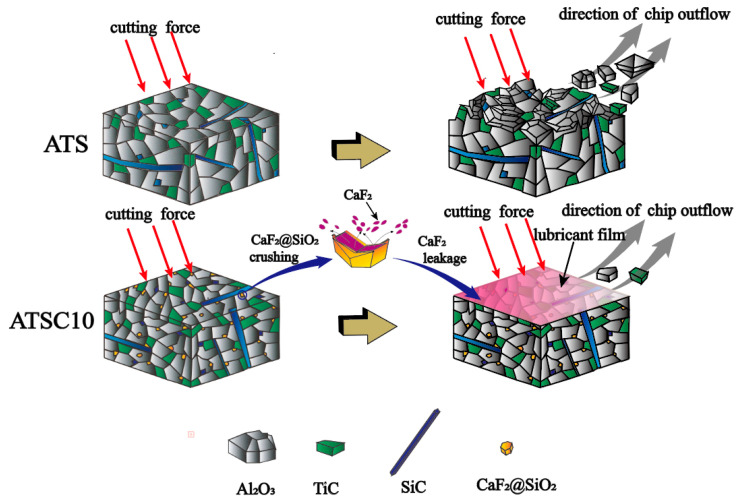
Comparison of the cutting mechanisms of ATS and ATSC10 cutting tools.

The mechanism by which the addition of nano-CaF_2_@SiO_2_ and SiC whiskers improves the cutting performance of the tools is discussed and analyzed below. The comparison of the microstructures of the two ceramic tools in Figure 1 shows that the particle size of the ATSC10 is significantly smaller than that of the ATS. Additionally, although the hardness of ATSC10 self-lubricating ceramic tools was lower than that of ATS ceramic tools, it was still high. At the same time, the ATSC10 self-lubricating ceramic tool had better flexural strength and fracture toughness. This endows ATSC10 ceramic tools with higher crack, crack propagation, and impact resistances, which is important in processing. Therefore, the addition of nano-CaF_2_@SiO_2_ and SiC whiskers can refine grains and strengthen the material, conferring the good mechanical properties of tools from ATSC10. During cutting, the ATS ceramic cutting tool generated a greater friction force between the cutter and workpiece, leading to gelling of the surfaces of the ceramic cutting tool and workpiece, resulting in the flow of the cutting tool material on the surface of the chip, as shown by the ATS ceramic cutting tool wear surface morphology before and after cutting. When cutting with the ATSC10 ceramic tool, the nano-coated particles were damaged, and CaF_2_, which has a low shear strength, was separated, forming a uniform and continuous lubricating film on the surface of the tool, thus reducing the cutting force and temperature during cutting. The ATSC10 ceramic tool should not easily crack from surface damage and reduced the generation of wear debris; the surface is easier to keep smooth. For this reason, the ATSC10 tools exhibited less wear and blade fracturing. Therefore, compared with the ATS ceramic cutting tool, the ATSC10 ceramic cutting tool had significantly lower cutting force and temperature, lower roughness, and better quality of the surface of the machined workpiece, and better mechanical properties of the tool itself. Figure 13 shows the cutting mechanism diagram for the ATS and ATSC10 tools.

**Figure 14:**
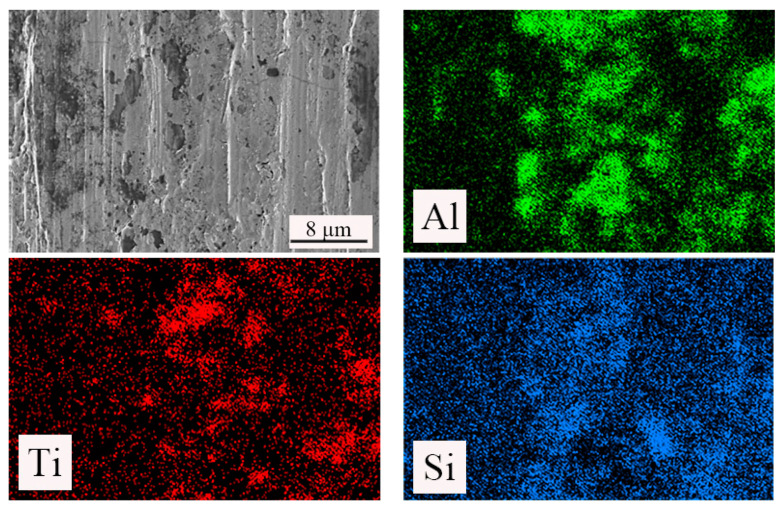

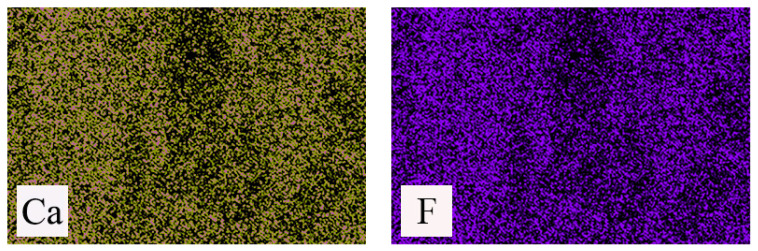
SEM image and corresponding EDS elemental mapping images (Al, Ti, Si, Ca, F) of flank wear of the ATSC10 cutting tool.

Figure 14 shows the SEM image and corresponding elemental mapping images. It can be seen that the elements of Si, Ca, and F are evenly distributed in the tool material. Considering that the Ca element and F element are from CaF_2_@SiO_2_, but Si is from CaF_2_@SiO_2_ and SiC, we found that the distribution of Ca and F was roughly the same and the distribution of Ca and F elements was denser and more extensive than that of Si elements. Therefore, we can say that in the process of cutting, CaF_2_ was separated, forming a uniform and continuous lubricating film on the surface of the tool, resulting in a denser and more extensive distribution of Ca and F elements than Si elements, which proves the formation of the CaF_2_ lubricating film.

## 4. Conclusions

In this study, adding nano-CaF_2_@SiO_2_ and SiC whiskers in ceramic tools for wear reduction and machinability improvement was studied. The main conclusions drawn are as follows.

(1) Al2O3/TiC ceramic tools with nano-coated CaF2@SiO2 and SiC whiskers were fabricated and tested in friction and wear experiments. According to the experimental data, the coefficient of friction of the ATSC10 self-lubricating ceramic tools first increased and then decreased, indicating that the coated solid lubricant had a good lubricating effect on the tools used in the experiments.

(2) The flank wear of the ATS and ATSC10 self-lubricating ceramic tools increased with increasing cutting speed, back-cutting rate, and feed rate. The surface roughness increased with an increase in the back-cutting and feed rates and decreased with an increase in the cutting speed. The service life of the ATSC10 self-lubricating ceramic tools was longer than that of the ATS ceramic tools; at the same time, the overall surface roughness of the ATSC10 self-lubricating ceramic tools is much lower than that of the ATS ceramic tools. The main cutting force of the ATSC10 ceramic tool with nanocoated particles and whiskers was 30% lower than that of the ATS ceramic tool. Compared with the ATS ceramic tool, the cutting temperature of the ATSC10 ceramic tool was 31.7% lower.

(3) The wear of the front surface of the ATS tool was mainly adhesive wear while that of the back tool surface was mainly abrasive wear. The face of the ATS ceramic tool has a clearly chipped edge. For ATSC10, the main forms of wear of the tool front surface were adhesive and abrasive wear, whereas that of the tool back surface was abrasive wear with slight adhesive wear. The addition of nano-coated particles and whiskers ensures that the ceramic tool maintains good cutting performance while having high mechanical properties.

## Figures and Tables

**Table 1:** Composition of ceramic cutting tool materials (vol.%).

Tool	Al_2_O_3_	TiC	MgO	Lubricant	SiC_w_
ATS	55.65	23.85	0.5	0	20
ATSC10	48.65	20.85	0.5	10	20

**Table 2:** The basic information of the raw material sheets.

Material	Phase	State	Particle Size
Al_2_O_3_	solid matter	crystalline form	0.5–1 μm
TiC	solid matter	crystalline form	0.5–1 μm
MgO	solid matter	crystalline form	0.5 μm
SiC	solid matter	crystalline form	0.5 um × 10 μm (diameter × length)
CaF_2_@SiO_2_	solid matter	amorphous form of SiO_2_ and crystalline form of CaF_2_	40–60 nm (SiO_2_ coating thickness is 10 nm)

**Table 3:** Mechanical properties of ceramic tool materials.

Material	Vickers Hardness (GPa)	Flexural Strength (MPa)	Fracture Toughness (MPa·m^1/2^)
ATS	17.67 ± 0.25	640 ± 20	4.97 ± 0.22
ATSC10	16.48 ± 0.20	714 ± 20	6.88 ± 0.22

**Table 4:** Geometrical parameters of the cutting tool.

Corner Radius *γ_ε_*	Rake Angle *γ*_0_	Relief Angle *α*_0_	Inclination Angle *λ_S_*	Cutting Edge Angle *κ_r_*	Chamfering Parameters *b_r_*_1_ *× γ_o_*_1_
0.2 mm	–5°	5°	0°	45°	0.2 mm × (–10°)
